# Eyesight and How to Care for It

**Published:** 1879-10

**Authors:** 


### BIBLIOGRAPHICAL.

*Eyesight, and How to Care for It.* By George C. Harlan, M. D., Surgeon to Wills' Eye Hospital, etc. Publishers: Lindsay & Blakiston, Philadelphia, 1879.

This small work, one of the series of the "American Health Primers," treats of a subject of the highest importance to the dental practitioner, and involves the sciences of anatomy, physiology and optics, as the intelligent care of the eyesight requires some knowledge, at least, of the structure and functions of the organ of vision. The author without claiming any originality, endeavors to popularize established facts and accepted theories, and after treating of the anatomy and physiology of the eye, refers to the more common injuries and diseases of this organ, ending with some of the most valuable practical suggestions in regard to the care such important organs demand, with rules for their safe exercise in reading, &c. The effects of school-life upon the sight also receives notice, and much useful information is given which cannot fail to be serviceable to every one who may peruse it.

